# Use of precision cut lung slices as a translational model for the study of lung biology

**DOI:** 10.1186/s12931-019-1131-x

**Published:** 2019-07-19

**Authors:** Guanghui Liu, Catherine Betts, Danen M. Cunoosamy, Per M. Åberg, Jorrit J. Hornberg, Kinga Balogh Sivars, Taylor S. Cohen

**Affiliations:** 1 RIA Safety, Clinical Pharmacology & Safety Sciences, BioPharmaceuticals R&D, AstraZeneca, Gothenburg, Sweden; 2 Pathology, Clinical Pharmacology & Safety Sciences, BioPharmaceuticals R&D, AstraZeneca, Cambridge, UK; 3 Bioscience, Respiratory Inflammation and Autoimmunity, BioPharmaceuticals R&D, AstraZeneca, Gothenburg, Sweden; 4 Microbial Sciences, BioPharmaceuticals R&D, AstraZeneca, One Medimmune Way, Gaithersburg, MD 20877 USA; 5 Present Address: Sanofi, Cambridge, MA USA

**Keywords:** Precision cut lung slices, Respiratory biology, Translational model

## Abstract

Animal models remain invaluable for study of respiratory diseases, however, translation of data generated in genetically homogeneous animals housed in a clean and well-controlled environment does not necessarily provide insight to the human disease situation. In vitro human systems such as air liquid interface (ALI) cultures and organ-on-a-chip models have attempted to bridge the divide between animal models and human patients. However, although 3D in nature, these models struggle to recreate the architecture and complex cellularity of the airways and parenchyma, and therefore cannot mimic the complex cell-cell interactions in the lung. To address this issue, lung slices have emerged as a useful ex vivo tool for studying the respiratory responses to inflammatory stimuli, infection, and novel drug compounds. This review covers the practicality of precision cut lung slice (PCLS) generation and benefits of this ex vivo culture system in modeling human lung biology and disease pathogenesis.

## Background

Respiratory disease is a leading cause of death, closely following heart disease, stroke and cancer according to the World Health Organization [1]. Despite large investment in research and development of treatments, incidence rates for major respiratory diseases, excluding cancer, such as chronic obstructive pulmonary disease (COPD), idiopathic pulmonary fibrosis, asthma and acute lung injury have remained constant or have increased over the last decade [2–4].

One factor for the limited success in identifying new drug therapies in many disease areas is the lack of translatable animal models used to evaluate efficacy and safety [5]. Mammalian model systems, primarily mice but also rats, pigs, guinea-pigs and rabbits have been invaluable in our quest towards a better understanding of respiratory function in health and disease. Next generation models such as the ferrets and pigs carrying the ∆F508 mutation in their CFTR gene come closer to mimicking human cystic fibrosis than any of the available murine models. However, there are still stark differences between the animal model phenotype and that observed clinically [6]. Thus, there is a need for alternatives to better translate between laboratory models and the clinic.

Historically, basic studies in respiratory research have primarily relied on transformed human cell lines, stimulated in culture and in isolation. More recently, culture systems comprising airway epithelial cells grown as a 2D monolayer or as a more complex 3D differentiated air liquid interface (ALI) culture including goblet, club and basal cells, have been mainstays of in vitro modeling [7–9]. Use of transwell systems enables development of a polarized epithelium with more natural alignment to respiratory surfaces, complete with ciliary beating and mucociliary clearance [10, 11]. Early advances in these models have involved the addition of immune cells to either the apical or basal chambers of the transwell system, enabling study of cellular crosstalk, and the differentiation of contact-dependent signaling versus secreted signaling factors [12–15]. Transwell systems, however, do not model the flow of air and liquid as would be experienced by the epithelium and endothelium in vivo. Microphysiological model systems were designed to overcome this limitation. Huh et al. first published on a single cell monolayer system in which cells are grown on an air liquid interface, beneath which culture media was pushed through one microfluidic system and above which either air or mucus was driven through a second fluidics system [16]. More recently, these “organ-on-a-chip” systems have been modified to include an endothelium, immune cells, and the mechanical flexibility to mimic the stretch of lung tissue during breathing [17].

Model systems such as those described above have pushed the boundaries of in vitro modeling capabilities, however, they still have drawbacks. Reliance on artificial scaffolds and the difficulty of acquiring primary cells from patients prevents such systems from completely recapitulating the complexities of respiratory disease. For structural relevance tracheal pieces in organ baths have been traditionally utilized for monitoring for smooth muscle function and constriction [18, 19]. For more mechanistic understanding and a system with capabilities to investigate multiple regions of the lung, Precision Cut Lung Slices (PCLS) are one potential solution. Slices can be cultured from explanted human lung, diseased human lung and animal models of disease. They contain all cell types found in the tissue of interest as well as accurately reflecting any changes to the underlying extracellular matrix associated with the disease. This review will focus on the use of PCLS for the study of respiratory disease, particularly regarding comparison to other translational models.

### Preparation and maintenance of PCLS

The concept of using tissue slices to study organ metabolism and toxicology was initiated in the 1920s, with substantial improvements in slicing technology since. Initially, liver slices were usually cut manually resulting in high variability of thickness and limited viability [20]. Stadie and Riggs made a significant step towards cutting tissue slices with a more consistent thickness in 1940s when they developed a microtome equipped with thin razor blade [21]. The use of a microtome reduced variability in thickness between slices to approximately 5%. Subsequent iterations of microtomes or tissue slicers are able to cut slices with enhanced precision and reproducible thickness, such slices became known as precision-cut tissue slices [22].

**Fig. 1:**
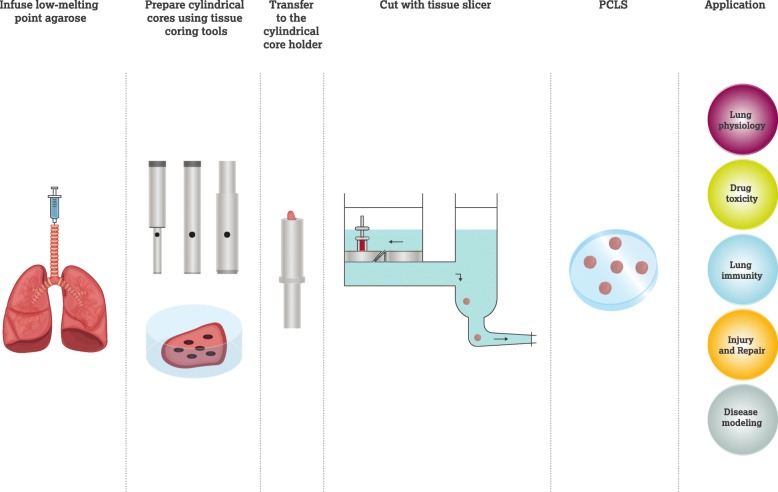
A schematic diagram showing the procedure to generate PCLS. After infusing agarose into the lung, cylindrical cores of the lung can be prepared by using specific tissue coring tools, followed by cutting with tissue slicer, generating lung slices with uniform diameter and thickness

For soft tissue such as lung, which is closely inter-networked by extracellular matrix and honeycombed to enable maximal gas exchange, it is technically challenging to obtain PCLS. However, a major breakthrough was achieved by Placke and Fisher in 1980s [23] by infusion of the airways of hamster and rat lungs with heated liquid agarose, which solidified at temperatures below 25 °C. The solidified agarose maintains the inflated state of the lung and prevents the collapse of the airways and delicate alveoli during slicing [23]. Recently the methodology used to prepare PCLS has been standardized (Fig. 1). The lung is inflated with warm buffer-equilibrated agarose solution via the trachea. Typically, agarose with a low-melting point is used in a concentration range of 0.5–3%. Once liquified at temperatures above 65 °C, agarose remains fluid when kept at 37 °C, only gelling when the temperature drops below 25 °C. Agarose is gently injected to avoid lung damage and minimize the inflation pressure, using an appropriate volume according to lung size. Furthermore, maintaining the animal on a 37 °C operation table or injecting the agarose solution at 40 °C will delay the gelling rate. After inflation, the lung is immersed in ice-cold buffer, initiating a rapid solidification of agarose, followed by precise cutting. In general, PCLS are prepared to a thickness of 100–500 μm. This process results in reproducible, uniform slices which can be deployed in a variety of ex vivo experimental protocols [24].

Additional challenges associated with the use of PCLS include maintaining the slices ex vivo. Typically, PCLS are submerged in culture medium in multi-well plates at 37 °C, 5% CO_2_ and 95–100% air humidity under tissue culture conditions, and medium is changed daily (examples of culture conditions shown in Table 1). The culture medium has been optimized to be supplemented with essential nutrients, enabling culture of viable PCLS for up to 14 days, as compared to previous reports of tissue surviving for only 3–5 days [26]. Furthermore, the addition of antibiotics such as penicillin and streptomycin prevent pathogen contamination from the onset of culture. Whilst in culture, PCLS remained viable and maintained normal metabolic activity, tissue homeostasis, structural integrity, and responses to stimulation with lipopolysaccharide (LPS) [26]. Although advanced culture techniques enhance PCLS survival, there are some changes in PCLS function during the culture period. For example, though human PCLS can constrict upon methacholine stimulus, LPS-induced TNF-α secretion, whilst maintained, can diminish over time [39]. Furthermore, PCLS lose certain cell populations, e.g. pneumocytes and lymphocyte cells and connective tissue fibers degrade during long-term cultivation, which may contribute to the decreased sensitivity of cultured PCLS in responses to external stimuli [27, 39]. In practice, PCLS can retain comparable viability and tissue homeostasis, either physiologically or functionally, during a cultivation period of 1 to 3 days, although extended periods can be achieved with optimized culture conditions [40].

**Table 1:** Representative use of PCLS as a translational model

Studies	PCLS origin	PCLS size	Culture conditions	Key findings	Reference
Wohlse et al.	Human donors	250-μm thickness, 9-mm diameter	MEM with supplements; 37 °C, 5% CO_2_, and 100% air humidity	•At least 3-day viability of PCLS •Immediate airway hyperresponsiveness of sensitized PCLS Inhibition of early allergic response of PCLS by blockade of leukotriene and thromboxane receptors	[25]
Temann et al.	Human donors	600-μm thickness, 8-mm diameter	DMEM/Ham’s F12; 37 °C, 5% CO_2_, 100% air humidity	•At least 14-day viability of PCLS with normal metabolic activity, tissue homeostasis and structural integrity •Pro-inflammatory responses of PCLS to LPS stimulation •Re-call immune responses of PCLS against seasonal influenza vaccine and tetanus toxoid	[26]
Bai et al.	Human donors	250-μm thickness, diameter not shown	DMEM/Ham’s F12; 37 °C, 5% CO_2_, 100% air humidity	•A long-term storage of PCLS by cryopreservation without affecting overall cell viability, functions of immune cells, and contraction and relaxation of in response to specific agonists and antagonists	[27]
Cooper et al.	Human donors	250-μm thickness, 8-mm diameter	Ham’s F12; 37 °C, 5% CO_2_, and 95% air humidity	•Attenuation of isoproterenol-induced relaxation of PCLS by albuterol •Reduction of cell-surface β_2_-adrenergic receptors by albuterol •Inhibition of albuterol-induced desensitization of β_2_-adrenergic receptors in PCLS by dexamethasone pretreatment	[28]
Alsafadi et al.	Human donors	500-μm thickness, diameter not shown	DMEM/Ham’s F12; 37 °C, 5% CO_2_, humidified	•Induction of early fibrosis-like changes in PCLS by using a combination of profibrotic growth factors and signaling molecules	[29]
Banerjee et al.	Human donors	250-μm thickness, 8-mm diameter	Ham’s F12; 37 °C, 5% CO_2_, and 95% air humidity	•Inhibition of the carbachol-induced PCLS contraction by trichostatin A •Inhibition of the agonist-induced PCLS contraction by trichostatin A via decreasing the agonist-induced mobilization of calcium in airway smooth muscle	[30]
Sturton et al.	Human donors, rat	270 ± 10-μm thickness, 8-mm diameter	RPMI 1640 for human PCLS and DMEM for rat PCLS; 37 °C, 5% CO_2_, and 95% air humidity	•Similarity of potency, intrinsic efficacy, and onset of action of indacaterol, formoterol and salmeterol to reverse the carbachol (for human) or serotonin (for rat)-induced contraction in both human and rat PCLS	[31]
Kennedy et al.	Asthma donors	250-μm thickness, diameter not shown	Ham’s F12; 37 °C, 5% CO_2_, and 95% air humidity	•Increased *il25*, *tslp*, and *il13* expression in asthma PCLS following RV39 infection •Increased carbachol-induced constriction in asthma PCLS after infection	[32]
Ghosh et al.	Asthma donors	350-μm thickness, 8-mm diameter	Ham’s F12; 37 °C, 5% CO_2_, and 95% air humidity	•Stimulation of NO-sGC-cGMP pathway bronchodilated PCLS from healthy donors •Chronic NO exposure caused sGC to show hallmarks of oxidative damage that observed in asthmatic human lung	[33]
Mercer et al.	IPF donors	250-μm thickness, 8-mm diameter	DMEM with supplements; 37 °C, 10% CO_2_, and 100% air humidity	•Active PI3K signalling within IPF fibrotic foci •PI3K/mTOR inhibitors reduced Akt phosphorylation in human IPF PCLS •PI3K/mTOR inhibitor reduced collagen formation markers in human IPF PCLS	[34]
Van Dijk et al.	Mouse	250-μm thickness, diameter not shown	DMEM with supplements; 37 °C, 5% CO_2_, and 95% air humidity	•A significant increase in mean linear intercept of elastase-treated PCLS ex vivo •Disorganized elastin and collagen fibers of elastase-treated PCLS •Decreased alveolar Type I and II marker expression of elastase-treated PCLS •Enhanced methacholine-induced airway narrowing and impaired chloroquine-induced airway opening of elastase-treated PCLS	[35]
Tatler et al.	Bleomycin-treated mouse	150-μm thickness, diameter not shown	DMEM; 37 °C, 5% CO_2_	•Significantly higher levels of collagen in PCLS from bleomycin-treated mouse •Caffeine significantly reduced collagen deposition over 5 days within bleomycin-PCLS	[36]
Henjakovic et al.	chemical allergen-sensitized mouse	220-μm thickness, diameter not shown	MEM with supplements; 37 °C, 5% CO_2_, and 100% air humidity	•High doses of TMA and DNCB induced cell dearh, tissue damage, and nuclear degeneration in naïve PCLS •TMA significantly decreased methacholine-induced bronchoconstriction	[37]
Lin et al.	Rat	300-μm thickness, diameter not shown	MEM; 37 °C incubator, 75% N_2_, 20% O_2_, 5% CO_2_, in scintillation vials on a bench roller	•CdCl_2_/TGF-β1-induced lung injury similar to that in early lung fibrogenesis in human	[38]

*MEM* Minimum Essential Medium, *DMEM* Dulbecco’s Modified Eagle Medium, *NO* nitric oxide, *sGC* soluble guanylate cyclase, *cGMP* cyclic guanosine monophosphate, *PI3K* Phosphoinositide 3-kinase, *mTOR* mammalian target of rapamycin, *TMA* trimellitic anhydride, *DNCB* 2,4-dinitrochlorobenzene

A further breakthrough in the use of PCLS came with the ability to store PCLS by cryopreservation for future use, which is particularly relevant for PCLS from rare patient populations [27, 41, 42]. Cryopreservation has minimal effects on overall cell viability, mitochondrial integrity and airway contraction in response to specific agonists and antagonists. Following cryopreservation, PCLS retain vital functions of immune cells, including phagocytosis and proliferation of lymphocytes. It is therefore now possible to store sections for prolonged periods, enabling collection of many donors/phenotypes prior to experimentation.

### PCLS studies in specific diseases

A key advantage of PCLS is its preservation of lung architecture. Morphologically, PCLS maintain relevant tissue structure, including small airways, respiratory parenchyma, structural and immune cell populations and connective tissue. In terms of cellular composition, PCLS retains most structural and immune cell organization [27, 43, 44]. Although PCLS with uniform thickness can be obtained, the number of specific cell types may vary from slice to slice based on regional variability within the lung, especially if there are disease related changes distributed non-uniformly. In principle, PCLS can be regarded as a “mini” lung in certain circumstances, albeit without a recruitable immune system, providing the unique opportunity to correlate cell-specific functions with organ physiology, as demonstrated by the complex response of PCLS to challenge and stimuli, e.g. airway contraction and immune responses [26, 42]. As a result, PCLS have been used as a model to evaluate asthma, COPD, idiopathic pulmonary fibrosis, allergy, infections as well as in toxicology studies [29, 35, 45–47]. Here, the most prominent respiratory diseases for which PCLS model systems have been utilized are summarized and translation to human disease is discussed (examples shown in Table 1).

### Asthma

Asthma is the most prevalent chronic respiratory disease, which affects more than 300 million people globally. It was the proposed cause of death of an estimated 400 thousand people in 2015 [48]. Clinically, asthma is characterized as an inflammatory disorder of the conducting airways, with traits such as airway inflammation, hyperresponsiveness, bronchoconstriction and airflow obstruction, but with a marked heterogeneity in both children and adults. There are two major categories of asthma, type 2 (T2) asthma and non-T2 asthma, which are defined by the presence or absence of a T2 immune response. T2 asthma is associated with sensitization to various allergens and infections, and infiltration of immune cells into airways [49–51]. Although various risk factors are related to asthma, it seems genetics and environmental factors synergistically contribute to its development. Genes such as *Ormdl3*, *Il1rl1/Il18r1*, *Hla-dq*, *Il33*, *Smad3* and *Il2rb9* that regulate epithelial barrier function and immune responses are associated with the occurrence of asthma in children and adults [52, 53]. Management of asthma has become more standardized according to guidelines recommended by the Global Initiative for Asthma (GINA), the US National Asthma Education and Prevention Program and the British Thoracic Society [54]. The treatment involves a stepwise strategy, by which patients are treated with low or high-dose inhaled corticosteroids, combined with other drugs such as leukotriene receptor antagonist and/or β_2_-receptor agonist, according to symptom severity and patient characteristics.

Animals have been successfully utilized to mimic various components in the pathophysiology of human asthma. Mice and rats are commonly used to replicate aspects of allergen induced asthma. The mouse develops relevant features of human allergic asthma upon allergic sensitization and challenge, resulting in allergen-specific response of T helper type 2 (T_H_2) cells, IgE increase, and airway hyperresponsiveness, bronchoconstriction and remodeling [55, 56]. Although great progress in understanding has been gained with mouse models of asthma, there are limitations to these models, particularly with translatability. Thus a system more closely representative of the human lung can provide further insight in disease understanding and for mechanistic investigations into drug development [57].

To improve translatability and disease understanding human PCLS from healthy and diseased patients have been utilized as an ex vivo tool to study asthma. PCLS obtained from healthy and diseased lungs demonstrate altered response to stimulation, including bronchoconstriction and hyperresponsiveness. These responses are aligned to those seen in patients as well as various animal models, suggesting a physiological mimicking of whole lung [25, 58, 59]. Moreover, PCLS from asthmatic patients show significantly enhanced airway inflammation and hyperresponsiveness following rhinovirus stimulation [32, 60]. Increased expression of genes, including *Il25*, *Tslp* and *Il13*, that are involved in asthma pathogenesis is detected in asthmatic human PCLS [61–63]. These observations correlating findings in asthmatic patients with PCLS models support PCLS as a promising system for asthma studies.

### COPD

COPD is a progressive inflammatory disease of the airways with irreversible limitation of airflow, associated with immune dysfunction and significant morbidity, mortality and healthcare costs [64, 65]. There are approximately 400 million people suffering from COPD and it is predicted to become the 3rd leading cause of death worldwide by 2030 [66]. In COPD patients, the importance of the immune components is well recognized in relation to an excessive inflammatory response to tobacco smoke and infection, leading to persistent pulmonary inflammation and acute exacerbations [64, 65, 67]. In contrast to asthma, the response to biologics (i.e. anti–IL-5, anti–IL-1β, anti–TNF-α and anti–IL-8) in COPD patients has been poor [68], highlighting the need for deep understanding of COPD mechanisms to apply the right treatment to the correct patient subset.

Although there are no in vivo models that encompass all aspects of the clinical disease pathology of COPD, some notable successes have been documented in animal models of cigarette smoke exposure, elastase-induced emphysema and LPS challenge [69]. Exposure of either guinea pigs or mice to cigarette smoke produces certain characteristics of human COPD, such as emphysema, small airway remodeling, and pulmonary hypertension [69, 70]. However, this model mimics only mild emphysema and usually takes months to develop [69]. Delivery of elastase to the lung rapidly induces emphysematic phenotype in mice, for which disease severity can be controlled by elastase dose, route of administration and duration [69, 71]. The physiological relevance of elastase and LPS models is questionable due to observed differences in mechanism [64, 69, 72].

The use of lung PCLS from in vivo models can be particularly beneficial for modelling COPD. For example, PCLS from smoke-exposed mice showed elevated expression of chemokines when stimulated with a viral mimic or influenza A virus [73]. Murine PCLS have been also used to demonstrate that Influenza A infection and cigarette smoke led to impairment of bronchodilator responsiveness to β2-adrenoceptor agonists [74]. Future studies using PCLS from COPD patients have the potential to enable both functional and phenotypic immune cell characterization, allowing greater integration of multiple analyses and better understanding of molecular mechanisms underlying disease heterogeneity.

### Idiopathic pulmonary fibrosis

Idiopathic pulmonary fibrosis (IPF) is a chronic lung disease pathologically characterized by progressive interstitial fibrosis, with an incidence of estimated 2.8–18 cases per 100000 people per year in Europe and North America [75, 76]. IPF patients typically experience shortness of breath, chronic dry cough, and nail clubbing. Currently, IPF is regarded as a consequence of multiple interacting environmental and genetic risk factors [77]. These factors, including cigarette smoking, older age, and mutations in genes associated with host defense, telomere maintenance and epithelial barrier function, convergently induce aberrant epithelial cell activation. This is followed by deposition of activated fibroblasts and myofibroblast promotion of lung fibrosis [78–80]. Specific cell signaling pathways, including transforming growth factor-beta (TGF-β), Wnt/β-catenin, vascular endothelial growth factor (VEGF) and phosphoinositide 3-kinase (PI3K-Akt), are thought to play a central role in the development of IPF [81]. As for other lung diseases, the use of animal models does not completely recapitulate human IPF. However, these models have contributed significantly to understanding of disease mechanisms. For example, the murine model of Bleomycin-induced lung fibrosis resembles early molecular signature of rapidly progressing IPF in human [82], and it has been used to evaluate antifibrotic effects of therapeutic agents [83, 84].

PCLS have been successfully used to study the early onset of lung fibrosis in IPF. By exposure to TGF-β1 and cadmium chloride, PCLS from human or rat have shown relevant pathohistological changes observed in early lung fibrosis, including upregulation of important pro-fibrotic genes, increased thickness of alveolar septa, and aberrant activation of pulmonary cells [38, 85, 86]. More recently, Alsafadi et al. established an ex vivo human PCLS model of early fibrosis, which requires exposure of PCLS to a combination of profibrotic growth factors and signaling molecules (TGF-β1, TNF-α, platelet-derived growth factor-AB, and lysophosphatidic acid), paving a way to study early-stage IPF pathomechanisms and evaluate novel therapies [29]. In addition, evaluation of novel therapies for IPF treatment using PCLS is currently underway. Caffeine, which inhibits TGF-β-induced increases of profibrotic gene expression, significantly reduces fibrosis in PCLS from bleomycin-treated mice [36]. Moreover, targeting PI3K signaling has been also shown to be a promising anti-fibrotic treatment strategy using IPF patient-derived PCLS [34].

### Infection and inflammation

The PCLS system has been used with some success to study innate responses to viral challenge, and to a lesser extent bacterial or bacterial component challenge. Use of the PCLS system has enabled a better understanding of which cells are infected in the intact lung as compared with in vitro air liquid interface cultures [87]. Goris et al. demonstrated different infectability of differentiated bovine airway epithelial cells, ALI grown cells and the upper epithelium in PCLS culture by bovine parainfluenza virus. This work demonstrated that in the PCLS system, infection was only observed in cells located beneath the epithelium and was most likely an artifact of the sliced tissue, indicating that when in the proper physiologic structure, the epithelium is resistant to infection. Comparable results were reported by Kirchhoff et al. [88]. These studies highlight the importance of studying cells in their physiologic environment with relevant cellular associations and structural architecture. Appropriate cell-cell interaction influences not only infectability, but also the response of the system to infection. For this reason, the PCLS system is a valuable tool in understanding the inflammatory response. From studies on the innate response to bacterial wall components such as LPS, to more complex mixed infection studies involving multiple viruses or viral and bacterial mixed infection, the PCLS system has enabled careful analysis of immune responses to each stimulus. In the most simplistic models, the PCLS system has been used to monitor the effect of LPS on the innate immune response, testing the effect of various immunomodulators on innate signaling [26, 89]. As discussed above, the ability to obtain slices from diseased lungs (e.g. COPD) provides a robust model system with which to study how respiratory disease influences infectivity and host responses. This is especially relevant to diseases such as COPD and asthma which are linked to pathogen induced exacerbation.

### Utility in efficacy and safety testing for new therapeutic targets

Regulatory toxicology testing is highly reliant on in vivo experiments in animals where it is key to identify organ toxicities that represent responses in a complex physiological environment. At present this is an essential step to identify and exclude risk in humans. In order to reduce candidate drug attrition due to toxicology or lack of clinical safety, predictive toxicology strategies have been implemented in earlier stages of the drug discovery process [90]. Such a proactive discovery safety strategy aims to timely identify and mitigate potential safety concerns related to the therapeutic target/concept or modality, and early signs indicate this contributes to increased pharmaceutical R&D productivity [91, 92]. This approach relies heavily on an arsenal of in vitro methods that enable investigational activities [93, 94]. Recent progress includes predictive cellular screening tools for hepatotoxicity, nephrotoxicity, cardiac toxicity and genetic toxicity [95–98]. For evaluation and mechanistic understanding of respiratory toxicity, a range of in vitro approaches have been used, where cellular-based systems have evolved to increasing complexity comprising primary human cells and with representation of different cell types [99].

In vitro systems such as ALI cultures have been applied to detect adverse impact of drugs in these more complex in vitro system, where the models comprise human primary epithelial cells on a basement membrane with a focus on predicting the toxic effects of inhaled drugs [100]. However, as described below the ALI models are still limited with regard to cell types, with no immune component or endothelial compartment, and hence do not fully replicate the architecture of the lung. As described above, microphysiological systems have been developed, introducing the vascular compartment as well as some aspects of lung dynamics, and although early in development some initial success in reproducing responses observed in vitro have been reported [16].

**Fig. 2:**
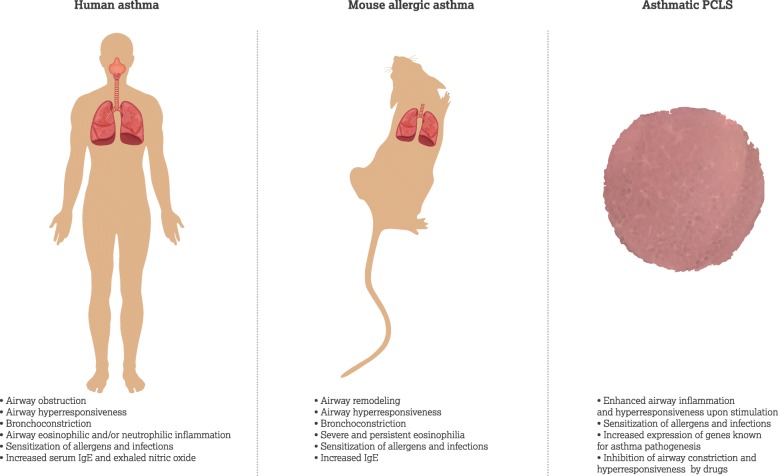
Translational studies of asthma using mouse model and PCLS. Asthmatic patient experiences airway obstruction and hyperresponsiveness, bronchoconstriction as well as airway inflammation (eosinophilic and/or neutrophilic). Mouse model of allergic asthma induced by ovalbumin or house dust mite mimics relevant features of human allergic asthma upon allergic sensitization and challenge, resulting in allergen-specific response of airways. Asthmatic PCLS obtained from mouse and patients show significantly enhanced airway inflammation and hyperresponsiveness following stimulation of allergens and infections

The more complex mix of cells and architecture in lung slice models is an attractive proposition for efficacy and toxicology testing, representing a translatable system between cell line or primary cell culture systems and the in vivo situation (Fig. 2). Indeed, PCLS can be a potential platform to investigate the efficacy and safety of drug candidates. In the context of asthma, inhaled corticosteroids control asthma symptoms by suppressing airway inflammation and consequentially reducing airway hyperresponsiveness, in combination with bronchodilators that specifically target signaling pathways or receptors critical in asthma pathogenesis [37, 101]. PCLS has been able to model this response, with glucocorticoids such as hydrocortisone, beclomethasone dipropionate and dexamethasone effectively abrogating airway constriction in PCLS from rat, horse and human induced by various bronchoconstrictors [28, 58, 102]. Similarly, several β_2_-receptor agonists have been shown to relax airway smooth muscle of contracted PCLS, mimicking their performance in animal models or in clinical trials [103–105].

PCLS have also been utilized to evaluate alternative targets for asthma treatment as increasing tolerance towards glucocorticoids and β_2_-receptor agonists necessitates development of novel therapeutic strategies. Targets relevant to asthma pathogenesis have therefore attracted more attention in recent years and investigations in PCLS have benefitted the evaluation of these targets as potential therapeutics. For example, inhibition of histone deacetylase by trichostatin A abrogates airway constriction in human PCLS and simultaneously inhibits airway hyperresponsiveness in antigen-challenged mice [30]. Furthermore, activation of soluble guanylate cyclase of airway smooth muscle by riociguat and cinaciguat analogs also triggers bronchodilation in normal human PCLS as well as reverses airway hyperresponsiveness in allergic asthmatic mice and restores normal lung function [33]. Indeed the use of PCLS in drug development is expanding with specific agonists or inhibitors targeting bitter-taste receptor, peroxisome proliferator activated receptor (PPAR) γ, Phosphoinositide-3 kinase (PI3K), BK channels and spleen tyrosine kinase (Syk) all undergoing investigation in PCLS [27, 106–109].

With regards to safety of inhaled therapeutics, key functional aspects and damage are assessed using well-validated pathological readouts on standardized safety studies intended for safety pharmacology assessments [110]. However, less well-defined is the impact on adverse impact on the immune system in the lung, particularly in response to the targeting of anti-inflammatory pathways and immune cell function with inherent risk to host defense. For many years the study of immunotoxicology in the lung has relied on the investigation of ex vivo lung tissues and fluids for cytokine release, basic histopathology to assess immune infiltrates and immune cell phenotyping by flow cytometry on BAL samples or lung tissue digests/disaggregations. Similarly, assessment of host defense in the presence of immunomodulatory drug candidates using preclinical models of lung viral and bacterial infection has been the mainstay of testing for increased infection risk. For this purpose, the type of study will comprise the species and pathogen of relevance to the disease under treatment and a druggable pathway of interest. However, these models are intensive, complex, cumbersome and expensive to undertake, with potential issues of translatability to the targeted human diseased population. The evolution of complex in vitro culture systems might provide a way forward for assessing adverse impact on host defense in a diseased population at an early point in the immunomodulatory drug development timeline. For example, ALI epithelial cultures supplemented with immune cells, and PCLS derived from diseased human lungs, where an immune resident cell component is present, could bridge the translation from preclinical testing to the patient population.

### Shortcomings of PCLS and alternatives

Although a lung slice comprises a section of lung tissue with the spatial arrangement and context preserved, the fact remains that tissue removed from the living organisms is to some extent fixed with regards to the cell populations contained within. Thus, a cultured PCLS represents a snapshot of cell populations currently resident in the lung tissue at time of excision but does not provide access to the wider, recruitable immune system, which is so critical in the whole organism. Additional consideration needs to be taken for heterogeneity in the lung. Differences in epithelial integrity, resident immune cell populations and responses to stimulation will be found between different locations within a single lobe. Furthermore, when studying diseased lung, heterogeneity between samples will further complicate data interpretation. Therefor appropriate power analysis should be used to account for slice to slice variability.

It is well documented that upon pathogenic invasion, the resident immune and tissue cells in the lung can release messenger molecules (cytokines and chemokines such as TNFα, IL-8, IL-6, CXCL1 and CXCL2) that attract further immune cells to the site to deal with the invading organism [111–113]. The timings and cell types recruited can vary by pathogen, but will likely include rapid recruitment of neutrophils, followed by a secondary wave of monocytes and lymphocytes all of which facilitate killing and removal of the pathogen [111, 114].

The inability to recruit non-resident immune cells, and viability of only 2 weeks, limits the extent to which the immune response in a cultured PCLS system can replicate the in vivo situation. However, the initiation and early signals induced by bacterial and viral pathogens can be studied in the PCLS model. This affords a wealth of opportunities to investigate the signals of the innate immune system and resident lung cells such as alveolar macrophages, alveolar type II cells and club cells. The lung slice model also provides an opportunity to investigate modification, by immunomodulatory treatments under investigation for inflammatory lung disease, of the initial innate immune signals released by resident lung cells upon exposure to pathogens. A challenge with any treatment of the PCLS system is the route of administration. Because the entire slice is bathed in the compound or stimulant of interest, one cannot directly translate treatment of the slice to inhaled or systemic application in vivo. Furthermore, translation of dosing from PCLS to human will prove difficult for the same reason. Despite this limitation, use of PCLS could provide great utility in both testing efficacy, but also in assessment of the potential adverse immunosuppressive impact of these drugs on host defense.

The PCLS model is typically used as a static system, not “breathing” as the lung typically would. This becomes important when modeling diseases such as ventilator induced lung injury, which rely on the mechanical stress applied by ventilation to damage the epithelial barrier [115]. A limited number of studies have tried to overcome this limitation by developing strategies to stretch, or deform, cultured slices. Davidovich et al. sutured the PCLS to a flexible membrane, which when pulled over a support uniformly stretched the tissue slice [116, 117]. Other groups have used air pressure to push the tissue slice in a bubble-like manner [118]. While these models mimic the effect of excessive ventilation, pressure is not applied via the epithelium, but rather transferred through the tissue via tension. It has yet to be determined if these models can accurately replicate the mechanical dynamics of ventilation. Alternative in vitro systems also comprising an immune component include structural lung cell compartment (epithelial and fibroblast cell lines) enhanced with primary human monocytes [119, 120], however, these organotypical systems also lack access to a recruitable cell population. The system most likely to mimic the ability of the lung to recruit cells into lung tissue in the longer term will be microphysiological systems whereby embedded lung cells of epithelial and/or endothelial origin have access to a fluidics system which may in future contain immune cells such as monocytes, neutrophils and T cells [121, 122]. Until the fluidics platforms evolve sufficiently, human PCLS afford the most translatable system for assessing both efficacy and safety issues of respiratory disease therapeutics.

## Conclusions

In vitro models of respiratory disease have matured over the past decades, changing from single cell systems to complex multidimensional, multicellular systems (Table 2). They are limited, however, in their ability to recreate the complexity of the lung, both in healthy and diseased states. PCLS models address this issue. By culturing sections of lungs from normal and diseased tissue across species, the PCLS model system is an alternative to traditional tissue culture, and a path towards reducing the number of animals used in the research of lung disease. There are areas of further development needed to progress the PCLS model to its full potential.

**Table 2:** Comparison of the different models used to study lung biology

Characteristics		ALI	Pulmonary organoids	PCLS	in vivo
Physical architecture	•Structural mimics	pseudostratified epithelium in vitro	3D multicellular tissue construct in vitro	ex vivo tissue system	in vivo disease models
	•Cell composition	predominated cell types are ciliated cells, goblet cells and basal cells	multiple differentiated cell types contain basal cells, multiciliated cells, secretory goblet cells and alveolar epithelial cells	all relevant cell types including structural and immune cells	all cell types
	•Cell origin	primary and immortalized bronchial epithelial cells	primary airway cells and human pluripotent stem cells	all resident cells	all resident and migratory cells
	•Recruitable immune cells	not available unless co-cultured	not available unless co-cultured	resident only	fully available
	•Extracellular matrix	not available	exogenous matrigel and hydrogels	endogenous	endogenous
Physiological function	•Physiological relevance	mucociliary differentiation; heterogeneous cell populations; epithelial barrier function; physiological responses to insults	morphological and functional mimicking of the airway; heterogeneous cell composition and spatial organization; self-renewal and differentiation	preservation of the lung architecture including small airways, respiratory parenchyma, cell populations and connective tissue; physiological responses to challenge and stimuli	intact lung; physiological responses to challenge and stimuli; mimicking of human respiratory diseases
	•Homeostasis	no	yes	yes	yes
	•Immune responses	epithelium related responses	epithelium related responses	collective responses from tissue components	whole lung responses including recruitable immune cells
	•Coculture potentials	yes, with fibroblasts and immune component	no data available	pre-existed multiple differentiated cell types	pre-existed multiple differentiated cell types

Methods for high-throughput processing and culture, improved drug delivery techniques to enable application of compounds to either the airway or blood stream, and incorporation of additional immune components will enhance this already powerful model. Despite their current drawbacks, PCLS are well situated to be a powerful model system for both basic research and drug screening workflows.

## Data Availability

Not applicable.

## Abbreviations

ALI: Air liquid interface
COPD: Chronic obstructive pulmonary disease
GINA: Global initiative for asthma
LPS: Lipopolysaccharide
PCLS: Precision cut lung slices
PI3K-Akt: Phosphoinositide 3-kinase
PPAR: Peroxisome proliferator activated receptor
Syk: Spleen tyrosine kinase
T2: Type 2
TGF-β: Transforming growth factor-beta
T_H_2: T helper type 2
VEGF: Vascular endothelial growth factor
